# Prospective evaluation of individualized vancomycin dosing based on a population pharmacokinetic model in patients with severe Gram-positive bacterial infection

**DOI:** 10.3389/fphar.2026.1852589

**Published:** 2026-06-25

**Authors:** Qile Xiao, Kexin Chen, Jian Qu, Hainan Zhang, Bohan Luo, Wei Wang, Chunyu Wang, Xiaomei Wu, Qihua Chen

**Affiliations:** 1 Department of Neurology, Second Xiangya Hospital, Central South University, Changsha, China; 2 Department of Pharmacy, Second Xiangya Hospital, Central South University, Changsha, China

**Keywords:** Gram-positive bacterial infection, individualized dosing, population pharmacokinetic modeling, severe infection, vancomycin

## Abstract

**Background:**

The population pharmacokinetic (PK) modeling of vancomycin has been validated to accurately predict concentration–time data in sepsis patients, but its effectiveness in clinical practice remains uncertain. This study aims to evaluate the clinical utility of individualized vancomycin dosing guided by a population PK model in patients with severe infections.

**Methods:**

This was a single-center, randomized, single-blind, controlled clinical trial. A total of 54 participants with severe infections caused by suspected or confirmed Gram-positive bacteria, who received vancomycin treatment during hospitalization, were prospectively included and identified as eligible. Participants were randomly allocated to either the individualized dosing group guided by a population PK model (n = 28) or the empirical dosing group (n = 26). After a 2-week treatment period, comparisons were made regarding pharmacokinetics/pharmacodynamics (PK/PD) target attainment, clinical efficacy, and safety between the two groups.

**Results:**

The individualized dosing group achieved an area under the concentration curve (AUC_24_) target rate of 69.23%, while the empirical dosing group had a rate of 8.33% (p = 0.000). Significant differences were also observed in body temperature, white blood cell count, Glasgow Coma Scale (GCS) score, and Acute Physiology and Chronic Health Evaluation (APACHE) II score after the 2-week treatment period between the two groups (p < 0.05). No significant difference in adverse events was found between the two groups.

**Conclusion:**

A daily dosage of individualized administration guided by a population PK model results in improved attainment rates for PK/PD targets and enhanced clinical efficacy without an increased risk of drug-related adverse reactions.

**Clinical Trial Registration:**

https://clinicaltrials.gov/study/NCT06161870, identifier NCT06161870.

## 1 Introduction

Neurocritically ill patients are highly susceptible to intracranial infections, severe pneumonia, bacteremia, and urinary tract infections as a consequence of common neurosurgical interventions and indwelling catheters (Lord et al., 2019; Erfani et al., 2022; Abulhasan et al., 2020). Epidemiological investigations conducted in developing countries have revealed a high prevalence of Gram-positive bacterial infections in neurointensive care units (NeuroICUs), particularly *Staphylococcus aureus*. Vancomycin has been extensively utilized as the preferred antibiotic for treatment of methicillin-resistant *S. aureus* (MRSA) and other Gram-positive bacteria (Tong et al., 2025; Holland et al., 2014). The management of severe infections poses ongoing challenges within the realm of antimicrobial therapy. Due to capillary leakage, inflammatory responses, and aggressive fluid loading, critically ill patients often encounter augmented renal clearance (ARC) and increased volume of distribution (Vd), resulting in elevated clearance rates for hydrophilic antibiotics such as vancomycin and subtherapeutic concentrations (Landersdorfer and Nation, 2021; Heffernan et al., 2021). For drugs that strongly bind to proteins, the effect of increased Vd and hypoproteinemia may lead to lower free concentrations in later dosing intervals, potentially compromising antibiotic efficacy (Landersdorfer and Nation, 2021; Heffernan et al., 2021; Asín-Prieto et al., 2015). Traditionally, clinicians assess renal function to promptly detect kidney dysfunction and adjust vancomycin dosing accordingly. However, the impact of ARC and increased Vd on the pharmacokinetics/pharmacodynamics (PK/PD) of vancomycin in critically ill patients has been overlooked. Actually, administration of conventional dosages to these patients may lead to suboptimal exposure, resulting in treatment failure or an elevated risk of adverse reactions (Heffernan et al., 2019; Cucci et al., 2020; Morbitzer et al., 2019a). ARC is prevalent among ICU patients (Bilbao-Meseguer et al., 2018). More importantly, recent studies have demonstrated a higher incidence of ARC among patients with intracranial infections (Lautrette et al., 2012; Blassmann et al., 2019; Chen et al., 2020), hemorrhagic strokes (Morbitzer et al., 2019b; May et al., 2015), and traumatic brain injuries (Udy et al., 2017; Dias et al., 2015) compared to mixed-cohort ICU patients.

According to the revised consensus guidelines for the treatment of serious MRSA infections, maintaining an area under the concentration curve (AUC_24_) within 400–600 mg h/L is necessary to achieve effective exposure for vancomycin, assuming a minimum inhibitory concentration (MIC) value of 1 mg/L. For patients with normal renal function and an MIC of 1 mg/L, an intermittent infusion of 15–20 mg/kg every 8–12 h is recommended (Rybak et al., 2020). However, these dosing recommendations do not account for critically ill patients with significant intra- and interindividual PK variability or those presenting with ARC. To minimize nephrotoxicity risk while attaining optimal PD targets, individualized antimicrobial dosing and population PK modeling are recommended (Alnezary et al., 2023; Dräg et al., 2025). In contrast to trough concentration (C_min_) monitoring, the population PK model relies less on the drug concentration at a specific time point. These models generate personalized estimates of the initial dose prior to obtaining the serum concentration, a process known as probabilistic dosing. This is based on covariates that significantly influence patients’ PK parameters. This approach improves the achievement of PD targets, even during the first administration (Gao et al., 2019; Roggeveen et al., 2022). The Vancomycin Calculator is an open-access advanced dosimetry software tool available online^1^. It incorporates a population PK model developed by Roberts JA et al., specifically tailored to sepsis patients, providing individualized dosing guidance at the bedside (Roberts et al., 2011). The model has been validated by multiple centers and demonstrates excellent predictive ability for concentration–time data in sepsis patients (Guo et al., 2019). Vancomycin clearance (CL_V_) is influenced by creatinine clearance (CLcr), while Vd is covariated by body weight (BW). Basic information, such as gender, age, BW, and CLcr, can be entered to calculate a dosing regimen expected to achieve the PD target^1^.

Evidence supporting administration based on population PK modeling primarily originates from PK/PD studies, with a lack of prospective, randomized controlled trial data to validate improvements in achieving PK/PD goals and clinical efficacy. Therefore, the primary objective of this study was to evaluate whether individualized vancomycin dosing guided by a population PK model is superior to empirical dosing in terms of attaining PK/PD targets and improving clinical efficacy in patients with severe Gram-positive bacterial infections.

## 2 Materials and methods

### 2.1 Ethical statement

This study was performed in line with the principles of the Declaration of Helsinki and complied with relevant Chinese laws and regulations. Approval was granted by the Ethics Committee of the Second Xiangya Hospital of Central South University (Approval No. Clinical study 2021.177). The privacy rights of human subjects were observed, and written informed consent was obtained from all individual participants or their authorized representatives. They signed informed consent regarding the publication of their data.

### 2.2 Trial design

This was a single-center, randomized, single-blind, controlled clinical trial prospectively designed and conducted from January 2021 to March 2024. A single-blind method was employed, in which the trial designer generated a random sequence and allocated participants to groups based on these numbers. Although the trial designers, investigators, and statistical analysts were aware of the participants’ group assignments, this information was not disclosed to the participants or their authorized representatives. Patient enrollment began in January 2021, following institutional ethics committee approval. This trial was retrospectively registered on ClinicalTrials.gov (NCT06161870, registered 7 December 2023) due to institutional practices at our center during the later stages of the COVID-19 pandemic, combined with limited awareness and administrative support for trial registration among clinician–investigators at that time, along with the inherently time-consuming nature of the registration process. Importantly, all study procedures, including data collection and analysis, were conducted according to the prespecified protocol approved by the Institutional Ethics Committee, with no amendments made after registration.

### 2.3 Participants

Participants were recruited from the NeuroICU of the Second Xiangya Hospital, Central South University; they had received vancomycin treatment for severe Gram-positive bacterial infection during hospitalization. Severe Gram-positive infection is characterized by bacteremia, sepsis, infective endocarditis, pneumonia, and encephalitis/meningitis caused by Gram-positive pathogens with clinical suspicion or microbiological culture evidence (Timsit et al., 2020). Participants were included based on the criteria outlined in Table 1, while those who discontinued treatment due to adverse reactions were excluded from the clinical efficacy analysis but were included in the safety evaluation for statistical purposes.

**TABLE 1:** Eligibility criteria.

Inclusion criteria	(1) Age ≥18 years (2) Participants with normal renal function (CLcr ≥80 mL/min/1.73 m^2^) or ARC (CLcr ≥130 mL/min/1.73 m^2^) (3) Fulfilling the etiological diagnosis or clinical diagnostic criteria for severe Gram-positive bacterial infection (4) Vancomycin administered as antimicrobial therapy during hospitalization
Exclusion criteria	(1) Pregnant or lactating women (2) Participants undergoing continuous renal replacement therapy (CRRT) after admission (3) Participants requesting termination of the trial and withdrawal of informed consent during the study

### 2.4 Intervention

For the individualized dosing group, the baseline demographic characteristics of the participants (gender, age, BW, and CLcr) were entered into the Vancomycin Calculator^1^ to obtain the predicted C_min_ and AUC_24_ values. The simulated dosing regimen expected to achieve the target AUC_24_ (400–600 mgh/L) (Rybak et al., 2020) was selected as the appropriate regimen. This calculator is based on the population pharmacokinetic model developed for critically ill patients with sepsis (Roberts et al., 2011).

For the empirical dosing group, vancomycin was administered intermittently at a dose of 15–20 mg/kg every 8–12 h, adjusted to actual BW.

After administering three consecutive doses of vancomycin, steady-state C_min_ levels were determined through blood sampling conducted half an hour prior to the administration of the fourth dose. Steady-state peak concentration (C_max_) levels were assessed by obtaining blood samples 1 hour after completion of the fourth dose. CL_V_, Vd, and AUC_24_ values were calculated for both the individualized and empirical dosing groups using a first-order PK equation (Pai et al., 2014) based on the measured concentrations.

Blood samples were centrifuged to separate the serum, and vancomycin concentrations (C_min_ and C_max_) were measured by high-performance liquid chromatography (HPLC) using a fully automatic two-dimensional liquid chromatography system (FLC-2801). Serum creatinine (SCr) levels were measured using an enzymatic method and an automatic biochemical analyzer. All assays were performed in the Clinical Laboratory of the Second Xiangya Hospital according to standard operating procedures and internal quality control protocols.

Demographic data, including gender, age, height, and BW, were collected. Microbiological cultures (e.g., blood, cerebrospinal fluid [CSF], sputum, or bronchoalveolar lavage fluid) were routinely collected prior to the initiation of vancomycin therapy according to the suspected infection site. The cultures were processed in the hospital’s clinical microbiology laboratory using standard procedures (VITEK 2 Compact system). Pathogen identification and antimicrobial susceptibility testing were performed, and the results were interpreted by clinicians to distinguish true infection from colonization or contamination. Data on antimicrobial agents administered within 72 h before vancomycin initiation were collected. Prior antibiotics were classified according to their activity against Gram-positive bacteria: agents with anti-Gram-positive activity (e.g., meropenem, piperacillin–tazobactam, cefoperazone–sulbactam, and oxacillin) versus those with limited activity (e.g., ceftriaxone, ceftazidime, and fluoroquinolones). Clinical data collection included SCr levels, CLcr, serum albumin levels, vancomycin dosing regimen, C_min_, C_max_, Vd, AUC_24_, baseline body temperature, white blood cell (WBC) count, neutrophil ratio (NEUT%), serum procalcitonin (PCT) levels, serum C-reactive protein (CRP) levels, Glasgow Coma Scale (GCS) score, Acute Physiology and Chronic Health Evaluation (APACHE) II score, and Sequential Organ Failure Assessment (SOFA) score. After 2 weeks of vancomycin treatment for both groups, the body temperature, WBC count, NEUT%, PCT levels, CRP levels, and GCS, APACHE II, and SOFA scores were recorded again. Additionally, NeuroICU length of stay, 30- and 90-day outcomes, drug-related adverse reactions, and concomitant use of nephrotoxic drugs were also documented. CLcr was calculated using the Cockcroft–Gault formula:

$$CLcr\;\left(mL/\min\right)=\frac{\left[\left(140-age\right)\times BW\;\left(kg\right)\right]}{\left[SCr\;\left(\mu mol/L\right)\times 0.818\right]}.$$

CLcr was calculated for female participants by multiplying the above formula by 0.85.

### 2.5 Outcome measures

The primary outcome measure aimed to compare the proportion of participants in both groups who achieved the PD target (AUC_24_ 400–600 mg h/L) after reaching a steady state following vancomycin administration.

The secondary outcome measures encompassed the assessment of APACHE II and SOFA scores after 2 weeks of vancomycin treatment and the determination of survival status (alive or dead) among participants on day 30 post-admission.

### 2.6 Definition of acute kidney injury (AKI)

Vancomycin-related adverse events, particularly AKI, were actively monitored throughout the treatment period and for 48 h after the last dose. AKI was defined according to the Kidney Disease: Improving Global Outcomes (KDIGO) criteria (Kidney Disease: Improving Global Outcomes KDIGO Acute Kidney Injury Work Group, 2012): an increase in SCr by ≥0.3 mg/dL (≥26.5 μmol/L) within 48 h or an increase in SCr to ≥1.5 times the baseline value, known or presumed to have occurred within the prior 7 days. All safety outcomes were evaluated by two independent investigators (a neurologist and a clinical pharmacist) using objective laboratory data and the predefined KDIGO criteria. No formal independent adjudication committee was established due to the single-center design and limited sample size of the study.

### 2.7 Statistical methods

Statistical analysis was performed using SPSS 26.0. Continuous data from normally distributed variables were presented as mean ± standard deviation (‾x ± s), and group comparisons were conducted using a Student’s t-test. For non-normally distributed variables, continuous data were presented as the median (first and third quartiles), and the Mann–Whitney U test, a non-parametric test, was used for group comparisons. Nominal data were expressed as either quantity or rate, with intergroup comparisons analyzed using a χ^2^ test. Statistical significance was defined as a two-sided p-value less than 0.05.

## 3 Results

**FIGURE 1:**
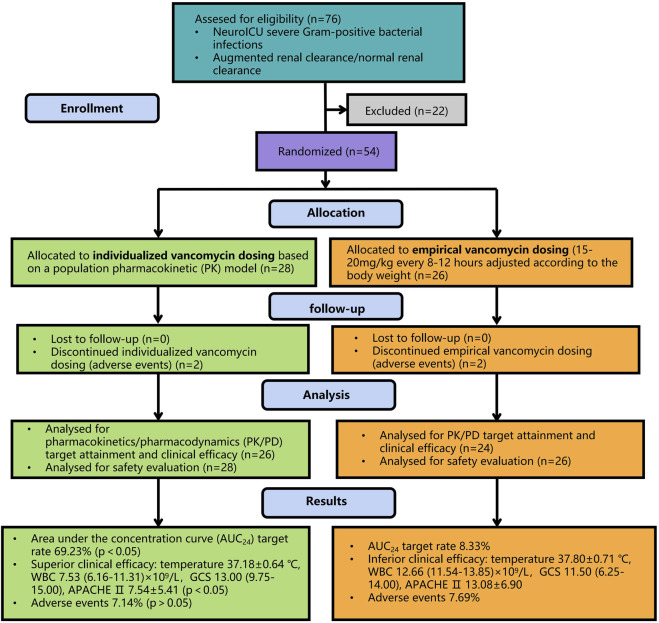
Enrollment, allocation, follow-up, data analysis, and main results of the study.

A total of 76 eligible adult patients were enrolled. Two of the subjects were pregnant. Eleven patients underwent continuous renal replacement therapy after admission. Nine patients declined to participate in this trial. Ultimately, 54 participants with severe infections caused by suspected or confirmed Gram-positive bacterial infection, who received vancomycin treatment during hospitalization, were prospectively included and identified as eligible. There were four patients who experienced drug-related adverse reactions during vancomycin treatment, and they were excluded from the analysis, except for safety evaluation purposes. Enrollment, allocation, follow-up, data analysis, and main results are shown in Figure 1.

### 3.1 Demographics, renal function, nutritional status, and infection site

A total of 50 participants with complete data were included in the study for all statistical analyses, with male participants accounting for 68.00% (34/50) and female participants accounting for 32.00% (16/50). The individualized dosing group comprised 26 cases, while the empirical dosing group consisted of 24 cases. No significant difference was observed in the proportion of ARC between the two groups (53.85% vs. 41.67%, p = 0.389). The CLcr for the individualized dosing group was measured at 131.50 (99.86–150.67) mL/min/1.73 m^2^, whereas it was recorded as 124.00 (109.21–154.75) mL/min/1.73 m^2^ for the empirical dosing group (p = 0.892). No statistically significant differences were found regarding demographics, renal function, nutritional status, or infection site between the two groups, as presented in Table 2.

**TABLE 2:** Demographics, renal function, nutritional status, and infection site in both groups.

Variable	Individualized dosing group (n = 26)	Empirical dosing group (n = 24)	Test statistics	P-value
Demographics				
Male, n (%)	18 (69.23%)	16 (66.67%)	0.038^c^	0.846
Age (years)	51.00 (38.75–58.25)	54.50 (45.25–61.75)	−0.943^a^	0.346
BW (kg)	66.40 ± 12.61	66.54 ± 11.10	−0.041^b^	0.968
Renal function				
ARC, n (%)	14 (53.85%)	10 (41.67%)	0.742^c^	0.389
CLcr (ml/min/1.73 m^2^)	131.50 (99.86–150.67)	124.00 (109.21–154.75)	−0.136^a^	0.892
Nutritional status				
Serum albumin (g/L)	35.65 ± 7.69	32.45 ± 5.11	1.719^b^	0.092
Infection site				
Bloodstream, n (%)	7 (26.92%)	8 (33.33%)	1.435^c^	0.507
Intracranial, n (%)	16 (61.54%)	11 (45.83%)		
Respiratory tract, n (%)	3 (11.54%)	5 (20.83%)		

^a^ Z-value.

^b^ t-value.

^c^ χ2-value.

### 3.2 Prior antimicrobial therapy and microbiological findings

Prior to vancomycin initiation, 45 out of 50 participants (90.00%) had received at least one course of alternative antimicrobial therapy. The most commonly used agents were ceftriaxone (n = 17, 34.00%), meropenem (n = 13, 26.00%), piperacillin–tazobactam (n = 11, 22.00%), and others (ceftazidime, cefoperazone–sulbactam, levofloxacin, etc.). Overall, 82.00% (41/50) of prior regimens included agents with anti-Gram-positive activity.

Microbiological cultures were obtained before vancomycin administration in all participants (100.00%). The culture positivity rate for clinically significant Gram-positive pathogens was 26.00% (13/50). Among the positive cases, *Staphylococcus aureus* (including one MRSA) was isolated in eight cases, coagulase-negative staphylococci in four cases, and *Enterococcus faecium* in one case. Gram-negative organisms (e.g., *Acinetobacter baumannii*, *Klebsiella pneumoniae*, and *Pseudomonas aeruginosa*) were frequently cultured from respiratory specimens, often representing colonization or co-infection. The relatively low positivity rate for Gram-positive pathogens was largely attributable to prior broad-spectrum antimicrobial exposure.

### 3.3 Baseline characteristics

Before initiating antimicrobial therapy, both groups of participants presented with fever, an elevated WBC count, NEUT%, and inflammatory indicators. There were no significant differences in baseline body temperature, WBC count, NEUT%, inflammatory markers, or disease severity between the two groups (p > 0.05), as indicated in Table 3.

**TABLE 3:** Baseline characteristics of the two groups.

Variable	Individualized dosing group (n = 26)	Empirical dosing group (n = 24)	Test statistics	P-value
Peripheral hemogram				
WBC count (×10^9^/L)	11.90 (8.30–14.60)	13.08 (10.70–16.22)	−0.738^a^	0.461
NEUT% (%)	85.16 ± 6.72	84.29 ± 5.57	0.494^b^	0.624
Inflammatory markers				
PCT (ng/mL)	0.22 (0.17–0.73)	0.19 (0.08–0.50)	−1.087^a^	0.277
CRP (mg/L)	79.95 ± 81.10	89.75 ± 88.07	−0.410^b^	0.684
Physiological measurement and disease severity				
Temperature (°C)	38.85 ± 0.70	38.80 ± 0.57	0.273^b^	0.786
GCS score	10.00 (6.75–15.00)	10.50 (6.00–13.00)	−0.537^a^	0.591
APACHE II score	19.50 (10.75–23.50)	14.50 (10.25–21.50)	−0.797^a^	0.425
SOFA score	4.19 ± 2.35	4.00 ± 3.26	0.241^b^	0.811

^a^ Z-value.

^b^ t-value.

### 3.4 Attainment of target PK/PD

The individualized dosing group received a higher total daily dosage of vancomycin compared to the empirical dosing group, with a dose of 3.00 (2.00–3.00) g/d for the former and a dose of 2.00 (2.00–2.00) g/d for the latter; this difference was statistically significant (p = 0.000).

The individualized dosing group demonstrated significantly higher values for AUC_24_ (439.58 ± 95.97 mgh/L vs. 304.21 ± 66.05 mgh/L, p = 0.000) and C_min_ (11.39 ± 3.46 mg/L vs. 8.03 ± 2.69 mg/L, p = 0.000) compared to the empirical dosing group. The individualized dosing group showed a significantly higher proportion of AUC_24_ reaching the PD target (AUC_24_ 400–600 mgh/L) (69.23% vs. 8.33%, p = 0.000), while there was no significant difference in the proportion of AUC_24_ >600 mgh/L (7.69% vs. 0.00%, p = 0.491), compared to the empirical dosing group. There were no statistically significant differences between the two groups regarding CL_V_ and Vd (p > 0.05), as shown in Table 4.

**TABLE 4:** Attainment of target PK/PD.

Variable	Individualized dosing group (n = 26)	Empirical dosing group (n = 24)	Test statistics	P-value
Total daily dose of vancomycin (g/d)	3.00 (2.00–3.00)	2.00 (2.00–2.00)	−4.112^a^	0.000^*^
C_min_ (mg/L)	11.39 ± 3.46	8.03 ± 2.69	3.806^b^	0.000^*^
C_max_ (mg/L)	25.09 ± 5.50	17.48 ± 4.23	5.451^b^	0.000^*^
AUC_24_ (mg·h/L)	439.58 ± 95.97	304.21 ± 66.05	5.762^b^	0.000^*^
AUC_24_ distribution				
<400 (mg·h/L)	6 (23.08%)	22 (91.67%)	23.828^c^	0.000^*^
400–600 (mg·h/L)	18 (69.23%)	2 (8.33%)	19.284^c^	0.000^*^
>600 (mg·h/L)	2 (7.69%)	0 (0.00%)	1.923^c^	0.491
CL_V_ (L/h)	6.45 (4.93–6.91)	7.12 (6.05–8.15)	−1.981^a^	0.054
Vd (L/kg)	0.70 (0.50–1.00)	0.85 (0.60–1.0)	−1.559^a^	0.119

Compared with the empirical dosing group, p* <0.05.

^a^ Z-value.

^b^ t-value.

^c^ χ2-value.

### 3.5 Clinical characteristics following a 2-week course of treatment

The individualized dosing group exhibited statistically significant differences in body temperature (37.18 °C ± 0.64 °C vs. 37.80 °C ± 0.71 °C) and WBC count [7.53 (6.16–11.31) ×10^9^/L vs. 12.66 (11.54–13.85) ×10^9^/L] compared to the empirical dosing group. After 2 weeks of treatment, a GCS score of 13.00 (9.75–15.00) was observed for the individualized dosing group, while the empirical dosing group had a score of 11.50 (6.25–14.00) (p = 0.048). The APACHE II score for the individualized dosing group was 7.54 ± 5.41, whereas it was 13.08 ± 6.90 (p = 0.003) for the empirical dosing group. No significant differences were observed between the two groups regarding the length of stay in the NeuroICU or outcomes at 30- and 90-day (p > 0.05) follow-ups, as shown in Table 5.

**TABLE 5:** Clinical characteristics of the participants following a 2-week course of treatment.

Variable	Individualized dosing group (n = 26)	Empirical dosing group (n = 24)	Test statistics	P-value
Peripheral hemogram and inflammatory markers				
WBC count (×10^9^/L)	7.53 (6.16–11.31)	12.66 (11.54–13.85)	−3.418^a^	0.000*
NEUT% (%)	77.25 (68.40–81.13)	81.95 (75.83–85.48)	−1.660^a^	0.097
PCT (ng/mL)	0.10 (0.08–0.20)	0.10 (0.06–0.22)	−0.583^a^	0.560
CRP (mg/L)	7.03 (3.55–20.93)	15.10 (5.47–47.50)	−1.621^a^	0.105
Physiological measurements and disease severity				
Temperature (°C)	37.18 ± 0.64	37.80 ± 0.71	−3.276^b^	0.002*
GCS score	13.00 (9.75–15.00)	11.50 (6.25–14.00)	−2.103^a^	0.048*
APACHE II score	7.54 ± 5.41	13.08 ± 6.90	−3.174^b^	0.003*
SOFA score	2.00 (0.75–3.00)	3.00 (1.00–4.75)	−1.890^a^	0.062
Clinical outcome				
NeuroICU length of stay (d)	13.50 (9.75–20.00)	14.00 (13.00–28.75)	−1.236^a^	0.217
30-day survival, n (%)	25 (96.15%)	24 (100.00%)	0.942^c^	1.000
90-day survival, n (%)	24 (92.31%)	23 (95.83%)	0.275^c^	1.000

Compared with the empirical dosing group, p* <0.05.

^a^ Z-value.

^b^ t-value.

^c^ χ2-value.

### 3.6 Safety evaluation

A total of four patients experienced drug-related adverse reactions during vancomycin treatment, and these events are fully reported in Table 6. These patients were excluded from the efficacy analysis (per-protocol efficacy population, n = 26 in the individualized dosing group and n = 24 in the empirical dosing group) but were included in the safety analysis (safety population, n = 28 in the individualized dosing group and n = 26 in the empirical dosing group). AKI occurred in one case in each group, where concomitant administration of other nephrotoxic agents (polymyxin B and furosemide) was also noted. Statistical analysis revealed no significant difference in the incidence of adverse reactions between the two groups (p > 0.05).

**TABLE 6:** Adverse events.

Variable	Individualized dosing group (n = 28)	Empirical dosing group (n = 26)	Test statistics	P-value
Leukopenia, n (%)	1 (3.57%)	1 (3.85%)	0.492^c^	1.000
AKI, n (%)	1 (3.85%)	1 (4.17%)		
No adverse events, n (%)	26 (92.86%)	24 (92.31%)		

^c^ χ2-value.

## 4 Discussion

### 4.1 Attainment of target PK/PD

In recent years, individualized vancomycin dosing based on population PK modeling has been preliminarily validated for accurately predicting PK/PD parameters in patients with severe infections. Among all the models evaluated with external datasets, the one-compartment linear model established by Roberts and collaborators for sepsis patients (Roberts et al., 2011) exhibited the lowest prediction error for vancomycin concentration–time data in multicenter ICU cohorts (Guo et al., 2019). However, there is a lack of reliable research confirming its optimization for antimicrobial therapy in clinical dosing practice. To the best of our knowledge, this study is the first clinical evaluation of a vancomycin population PK model for patients with severe Gram-positive bacterial infections in NeuroICUs.

ICU patients exhibit significant PK/PD variability (Abraham et al., 2019). Accurate CL_V_ and Vd values were calculated using C_min_ and C_max_ values obtained from blood samples in our study, following the first-order PK equation. Our results revealed significantly elevated CL_V_ (6.68 ± 1.41 L/h) and Vd (0.77 ± 0.25 L/kg) values, surpassing those mentioned in previous literature reports, which ranged from 2.97 L/h to 4.58 L/h for CL_V_ and 0.3 L/kg to 0.45 L/kg for Vd (Roberts et al., 2011; Pongchaidecha et al., 2020), suggesting that patients with severe infections, normal renal function and ARC experience significant PK changes. Accurately predicting the PK/PD of vancomycin is crucial for achieving optimal drug exposure (Cunio et al., 2020). systemic inflammatory response syndrome (SIRS) and increased renal perfusion contribute to ARC and increased CL_V_, which directly impact serum concentration (Tesfamariam et al., 2024). In addition, capillary leakage resulting from sepsis, shock, hypoproteinemia, and the necessary fluid resuscitation can significantly increase Vd, leading to inadequate blood concentrations (Tanaka, 2025), explaining why patients with severe infections with normal renal function are also prone to suboptimal drug exposure. In fact, a series of observational studies has demonstrated that it is challenging to attain PD targets when administering standard doses of vancomycin to critically ill patients, and this suboptimal drug exposure is particularly pronounced in ARC patients (Villanueva et al., 2019; Zhao et al., 2022; He et al., 2020).

In this trial, we employed the model developed by Roberts et al. (2011) to estimate individual CL_V_ and Vd in the individualized dosing group, which lacked initial plasma concentration data. The individualized dosing group received a significantly higher daily dose of vancomycin compared to the empirical dosing group (p = 0.000), which underwent standard dosing. The AUC_24_ in the individualized dosing group was significantly higher (p = 0.000), and there was a significant increase in the proportion of AUC_24_ reaching the PD target (p = 0.000), with no observed increase in the proportion of AUC_24_ > 600 mg h/L. The data above indicated that compared with the standard dose, intermittent vancomycin infusion guided by a population PK model increased the proportion of attainment of satisfactory PK/PD exposure for severely infected patients who have experienced significant PK changes, even without obtaining the initial blood concentration.

Although the AUC_24_ target of 400–600 mg h/L is primarily established and validated for serious MRSA infections based on an MIC of 1 mg/L (Rybak et al., 2020), its applicability to non-MRSA Gram-positive infections (such as infections caused by coagulase-negative staphylococci or *Enterococcus*) remains less well defined. In the present study, while the majority of culture-positive Gram-positive pathogens were *Staphylococcus aureus*, a proportion involved other organisms, including coagulase-negative staphylococci and *Enterococcus faecium*. Emerging evidence supports the potential utility of similar AUC targets for non-MRSA Gram-positive infections. Recent studies in patients with enterococcal bacteremia have demonstrated that higher vancomycin AUC_24_/MIC ratios (AUC_24_/MIC ≥389) (Tochikura et al., 2024) or the achievement of AUC targets of approximately 400–616 μg h/mL (Sel et al., 2025) are associated with improved clinical outcomes. These findings suggest that individualized dosing guided by population PK modeling to attain the AUC_24_ target of 400–600 mg h/L may also confer clinical benefits in a broader spectrum of Gram-positive infections encountered in clinical practice. However, further prospective studies are warranted to confirm the optimal PK/PD targets specifically for non-MRSA pathogens.

### 4.2 Efficacy of antibacterial therapy and safety evaluation

In this trial, 90.00% of participants had received broad-spectrum antimicrobial therapy prior to vancomycin initiation, with 82.00% of regimens providing coverage against Gram-positive organisms. This extensive prior exposure largely explains the relatively low culture positivity rate for significant Gram-positive pathogens (26.00%). Therefore, microbiological clearance was not adopted as an efficacy evaluation indicator. Instead, clinical efficacy was assessed using objective parameters (body temperature, WBC count, GCS score, APACHE II score, and so on). Significant differences were observed in body temperature, WBC count, GCS score, and APACHE II score between the two groups following a 2-week treatment period (p < 0.05). The findings suggest that individualized antibacterial therapy demonstrates superior clinical efficacy compared to empirical therapy in terms of anti-inflammatory effects and reduction in disease severity. Recently, a meta-analysis demonstrated that individualized antimicrobial dosing is associated with a significant decrease in treatment failure. Enhancement in microbiological outcomes was also observed, although the difference was not significant (Sanz-Codina et al., 2023).

A retrospective, multicenter cohort study examining patients receiving vancomycin therapy for severe MRSA infections revealed a significant correlation between an AUC_24_/MIC of <505 mg h/L and 30-day mortality (Hanai et al., 2024). However, in a prospective, multicenter, observational study of patients with MRSA bacteremia treated with vancomycin, higher vancomycin exposures did not confer a lower treatment failure risk (defined as 30-day mortality or persistent bacteremia ≥7 days) but were associated with more AKI (Lodise et al., 2020). In this trial, we did not observe a significant difference in outcomes (length of NeuroICU stay and 30- and 90-day outcomes) between the two groups, with significantly different AUC_24_ as well, potentially attributed to various factors. First, the statistical results may have been influenced by the low proportion of participants who died, as reflected in the 30- and 90-day outcome variables. Second, participants treated with multiple antimicrobials could potentially attenuate any negative effects of vancomycin subexposure on outcomes. Finally, it is plausible that achieving target PD may not be indispensable for a better prognosis, as bacterial susceptibility, organ dysfunction, severity of brain injury, and other NeuroICU interventions could also impact patient outcomes.

A concern associated with the utilization of high doses of vancomycin is that renal toxicity escalates proportionally with increased drug exposure (Tsutsuura et al., 2021). AUC-guided dosing strategies had fewer cases of vancomycin-induced AKI than the trough-guided dosing strategies (Abdelmessih et al., 2022). AUC ≥700 mg h/L was significantly associated with an increased AKI risk (Tangvichitrerk et al., 2025). In this trial, one case of AKI was observed in both the individualized and empirical dosing groups, with no statistically significant difference in the incidence. Additionally, there was no significant difference in the proportion of participants exhibiting an AUC_24_ ≥600 mg h/L. The AUC_24_ values for all participants remained consistently below 700  mg h/L. Furthermore, we did not observe any differences in other adverse events between the two groups. The findings demonstrate that individualized antibacterial treatment results in an augmentation of the vancomycin exposure target and clinical efficacy, without a concomitant escalation in drug toxicity incidence. It is noteworthy that participants in both the individualized and empirical dosing groups who developed AKI were co-administered with other nephrotoxic drugs, along with vancomycin. Concurrent usage of nephrotoxic medications can exacerbate the nephrotoxicity associated with vancomycin (Kim et al., 2022). It is advisable to minimize the concurrent administration of nephrotoxic drugs in these individuals.

### 4.3 Limitations

This study has several limitations. First, due to the retrospective registration of this trial, the possibility of selective reporting bias cannot be entirely excluded. However, all outcomes and analyses were prespecified in the ethics-approved protocol. Second, this single-center study with a small sample size may limit the robustness of the results. The final sample size was smaller than originally planned, primarily due to high refusal rates by families concerned about off-label dosing and the exclusion of patients requiring CRRT. These factors reflect the real-world challenges of conducting trials in neurocritically ill populations. Third, the CLcr calculated using the Cockcroft–Gault equation tends to be lower and less accurate than that measured by urine collection, potentially leading to an underestimation of the glomerular filtration rate (Monteiro et al., 2023). Fourth, due to prior antimicrobial treatments received by a significant proportion of participants before enrollment, resulting in low rates of positive microbial detection, microbial clearance was not used for efficacy evaluation. Finally, we did not consider the influence of vancomycin CSF concentration on the dosing strategy for patients with central nervous system (CNS) infection. A series of recent studies on the PK/PD characteristics of vancomycin in CSF have demonstrated that serum concentration, blood–brain barrier disruption, and the daily CSF drainage amount are the main factors affecting the CSF concentrations (Xiao et al., 2022). However, due to the complexity of the factors affecting the PK/PD of drugs in the CSF, determining the effective CSF exposure target and the dosing regimen required to achieve it remains challenging. Future research endeavors entail designing a larger, randomized controlled trial with an expanded sample size to facilitate more comprehensive investigations into the impact of PK/PD target attainment on clinical efficacy among patients with severe infections.

## 5 Conclusion

Patients with severe infections with normal renal function and ARC exhibited significant PK/PD alterations, characterized by a substantial increase in both CL_V_ and Vd. A daily dose of individualized vancomycin guided by a population PK model is higher than an empirically determined dose, resulting in better attainment rates of PK/PD targets and improved clinical efficacy without increasing the risk of drug-related adverse reactions.

## Data Availability

The raw data supporting the conclusions of this article will be made available by the authors, without undue reservation.

## Footnotes

1 Kane SP (2025). Vancomycin Calculator. https://clincalc.com/Vancomycin [Accessed 8 April 2026].

## References

[B1] Abdelmessih E. Patel N. Vekaria J. Crovetto B. SanFilippo S. Adams C. (2022). Vancomycin area under the curve versus trough only guided dosing and the risk of acute kidney injury: systematic review and meta-analysis. Pharmacotherapy 42 (9), 741–753. 10.1002/phar.2722 35869689 PMC9481691

[B2] Abraham J. Sinnollareddy M. G. Roberts M. S. Williams P. Peake S. L. Lipman J. (2019). Plasma and interstitial fluid population pharmacokinetics of vancomycin in critically ill patients with sepsis. Int. J. Antimicrob. Agents 53 (2), 137–142. 10.1016/j.ijantimicag.2018.09.021 30296581

[B3] Abulhasan Y. B. Abdullah A. A. Shetty S. A. Ramadan M. A. Yousef W. Mokaddas E. M. (2020). Health care-associated infections in a neurocritical care unit of a developing country. Neurocrit Care 32 (3), 836–846. 10.1007/s12028-019-00856-8 31562598

[B4] Alnezary F. S. Almutairi M. S. Gonzales-Luna A. J. Thabit A. K. (2023). The significance of Bayesian pharmacokinetics in dosing for critically ill patients: a primer for Clinicians using vancomycin as an example. Antibiot. (Basel) 12 (9), 1441. 10.3390/antibiotics12091441 37760737 PMC10525617

[B5] Asín-Prieto E. Rodríguez-Gascón A. Isla A. (2015). Applications of the pharmacokinetic/pharmacodynamic (PK/PD) analysis of antimicrobial agents. J. Infect. Chemother. 21 (5), 319–329. 10.1016/j.jiac.2015.02.001 25737147

[B6] Bilbao-Meseguer I. Rodríguez-Gascón A. Barrasa H. Isla A. Solinís M. Á. (2018). Augmented renal clearance in critically ill patients: a systematic review. Clin. Pharmacokinet. 57 (9), 1107–1121. 10.1007/s40262-018-0636-7 29441476

[B7] Blassmann U. Hope W. Roehr A. C. Frey O. R. Vetter-Kerkhoff C. Thon N. (2019). CSF penetration of vancomycin in critical care patients with proven or suspected ventriculitis: a prospective observational study. J. Antimicrob. Chemother. 74 (4), 991–996. 10.1093/jac/dky543 30689877

[B8] Chen Y. Liu L. Zhu M. (2020). Effect of augmented renal clearance on the therapeutic drug monitoring of vancomycin in patients after neurosurgery. J. Int. Med. Res. 48 (10), 300060520949076. 10.1177/0300060520949076 33100081 PMC7604945

[B9] Cucci M. Wooten C. Fowler M. Mallat A. Hieb N. Mullen C. (2020). Incidence and risk factors associated with multi-drug-resistant pathogens in a critically ill trauma population: a retrospective cohort study. Surg. Infect. (Larchmt) 21 (1), 15–22. 10.1089/sur.2019.031 31210580

[B10] Cunio C. B. Uster D. W. Carland J. E. Buscher H. Liu Z. Brett J. (2020). Towards precision dosing of vancomycin in critically ill patients: an evaluation of the predictive performance of pharmacometric models in ICU patients. Clin. Microbiol. Infect. S1198-743X (20), 30388. 10.1016/j.cmi.2020.07.005 32673799

[B11] Dias C. Gaio A. R. Monteiro E. Barbosa S. Cerejo A. Donnelly J. (2015). Kidney-brain link in traumatic brain injury patients? A preliminary report. Neurocrit Care 22 (2), 192–201. 10.1007/s12028-014-0045-1 25273515

[B12] Dräger S. Minichmayr I. K. Alipanah-Lechner N. Barreto E. F. Bos L. D. J. Fleuren L. M. (2025). Dose individualisation of antibiotics in critically ill patients with inflammation: a narrative review. Br. J. Clin. Pharmacol. 91 (11), 3042–3053. 10.1002/bcp.70185 40813897 PMC12569563

[B13] Erfani Z. Jelodari M. H. Rawling J. A. Eajazi A. Deever D. Mirmoeeni S. (2022). Pneumonia in nervous system injuries: an analytic review of literature and recommendations. Cureus 14 (6), e25616. 10.7759/cureus.25616 35784955 PMC9249029

[B14] Gao Y. Hennig S. Barras M. (2019). Monitoring of tobramycin exposure: what is the best estimation method and sampling time for clinical practice? Clin. Pharmacokinet. 58 (3), 389–399. 10.1007/s40262-018-0707-9 30140975

[B15] Guo T. van Hest R. M. Roggeveen L. F. Fleuren L. M. Thoral P. J. Bosman R. J. (2019). External evaluation of population pharmacokinetic models of vancomycin in large cohorts of intensive care unit patients. Antimicrob. Agents Chemother. 63 (5), e02543. 10.1128/AAC.02543-18 30833424 PMC6496102

[B16] Hanai Y. Hashi H. Hanawa K. Endo A. Miyazaki T. Yamaguchi T. (2024). Predictive value of vancomycin AUC_24_/MIC ratio for 30-day mortality in patients with severe or complicated methicillin-resistant *Staphylococcus aureus* infections: a multicenter retrospective study. Pharm. Res. 41 (7), 1381–1389. 10.1007/s11095-024-03728-9 38886259

[B17] He J. Yang Z. T. Qian X. Zhao B. Mao E. Q. Chen E. Z. (2020). A higher dose of vancomycin is needed in critically ill patients with augmented renal clearance. Transl. Androl. Urol. 9 (5), 2166–2171. 10.21037/tau-20-1048 33209680 PMC7658164

[B18] Heffernan A. J. Germano A. Sime F. B. Roberts J. A. Kimura E. (2019). Vancomycin population pharmacokinetics for adult patients with sepsis or septic shock: are current dosing regimens sufficient? Eur. J. Clin. Pharmacol. 75 (9), 1219–1226. 10.1007/s00228-019-02694-1 31154476

[B19] Heffernan A. J. Mohd S. L. S. Lipman J. Roberts J. A. (2021). A personalised approach to antibiotic pharmacokinetics and pharmacodynamics in critically ill patients. Anaesth. Crit. Care Pain Med. 40 (6), 100970. 10.1016/j.accpm.2021.100970 34728411

[B20] Holland T. L. Arnold C. Fowler V. G. Jr. (2014). Clinical management of *Staphylococcus aureus* bacteremia: a review. JAMA 312 (13), 1330–1341. 10.1001/jama.2014.9743 25268440 PMC4263314

[B21] Kidney Disease: Improving Global Outcomes (KDIGO) Acute Kidney Injury Work Group (2012). KDIGO clinical practice guideline for acute kidney injury. Kidney Int. Suppl. 2 (1), 1–138. 10.1038/kisup.2012.1

[B22] Kim J. Y. Yee J. Yoon H. Y. Han J. M. Gwak H. S. (2022). Risk factors for vancomycin-associated acute kidney injury: a systematic review and meta-analysis. Br. J. Clin. Pharmacol. 88 (9), 3977–3989. 10.1111/bcp.15429 35665530

[B23] Landersdorfer C. B. Nation R. L. (2021). Key challenges in providing effective antibiotic therapy for critically ill patients with bacterial sepsis and septic shock. Clin. Pharmacol. Ther. 109 (4), 892–904. 10.1002/cpt.2203 33570163

[B24] Lautrette A. Phan T. N. Ouchchane L. Aithssain A. Tixier V. Heng A. E. (2012). High creatinine clearance in critically ill patients with community-acquired acute infectious meningitis. BMC Nephrol. 13, 124. 10.1186/1471-2369-13-124 23013403 PMC3502432

[B25] Lodise T. P. Rosenkranz S. L. Finnemeyer M. Evans S. Sims M. Zervos M. J. (2020). The emperor's new clothes: prospective observational evaluation of the association between initial VancomycIn exposure and failure rates among ADult HospitalizEd patients with methicillin-resistant *Staphylococcus aureus* bloodstream infections (PROVIDE). Clin. Infect. Dis. 70 (8), 1536–1545. 10.1093/cid/ciz460 31157370 PMC7145993

[B26] Lord A. S. Nicholson J. Lewis A. (2019). Infection prevention in the neurointensive care unit: a systematic review. Neurocrit Care 31 (1), 196–210. 10.1007/s12028-018-0568-y 29998427 PMC6329681

[B27] May C. C. Arora S. Parli S. E. Fraser J. F. Bastin M. T. Cook A. M. (2015). Augmented renal clearance in patients with subarachnoid hemorrhage. Neurocrit Care 23 (3), 374–379. 10.1007/s12028-015-0127-8 25761425 PMC12968823

[B28] Monteiro E. Fraga Pereira M. Barroso I. Dias C. C. Czosnyka M. Paiva J. A. (2023). Creatinine clearance in acute brain injury: a comparison of methods. Neurocrit Care 39 (2), 514–521. 10.1007/s12028-023-01714-4 37016059

[B29] Morbitzer K. A. Rhoney D. H. Dehne K. A. Jordan J. D. (2019a). Enhanced renal clearance and impact on vancomycin pharmacokinetic parameters in patients with hemorrhagic stroke. J. Intensive Care 7, 51. 10.1186/s40560-019-0408-y 31832200 PMC6868795

[B30] Morbitzer K. A. Jordan J. D. Dehne K. A. Durr E. A. Olm-Shipman C. M. Rhoney D. H. (2019b). Enhanced renal clearance in patients with hemorrhagic stroke. Crit. Care Med. 47 (6), 800–808. 10.1097/CCM.0000000000003716 30870191

[B31] Pai M. P. Neely M. Rodvold K. A. Lodise T. P. (2014). Innovative approaches to optimizing the delivery of vancomycin in individual patients. Adv. Drug Deliv. Rev. 77, 50–57. 10.1016/j.addr.2014.05.016 24910345

[B32] Pongchaidecha M. Changpradub D. Bannalung K. Seejuntra K. Thongmee S. Unnual A. (2020). Vancomycin area under the curve and pharmacokinetic parameters during the first 24 hours of treatment in critically ill patients using Bayesian forecasting. Infect. Chemother. 52 (4), 573–582. 10.3947/ic.2020.52.4.573 33263245 PMC7779987

[B33] Roberts J. A. Taccone F. S. Udy A. A. Vincent J. L. Jacobs F. Lipman J. (2011). Vancomycin dosing in critically ill patients: robust methods for improved continuous-infusion regimens. Antimicrob. Agents Chemother. 55 (6), 2704–2709. 10.1128/AAC.01708-10 21402850 PMC3101407

[B34] Roggeveen L. F. Guo T. Fleuren L. M. Driessen R. Thoral P. van Hest R. M. (2022). Right dose, right now: bedside, real-time, data-driven, and personalised antibiotic dosing in critically ill patients with sepsis or septic shock-a two-centre randomised clinical trial. Crit. Care 26 (1), 265. 10.1186/s13054-022-04098-7 36064438 PMC9443636

[B35] Rybak M. J. Le J. Lodise T. P. Levine D. P. Bradley J. S. Liu C. (2020). Therapeutic monitoring of vancomycin for serious methicillin-resistant *Staphylococcus aureus* infections: a revised consensus guideline and review by the American society of health-system pharmacists, the infectious diseases society of America, the pediatric infectious diseases society, and the society of infectious diseases pharmacists. Am. J. Health Syst. Pharm. 77 (11), 835–864. 10.1093/ajhp/zxaa036 32191793

[B36] Sanz-Codina M. Bozkir H. Ö. Jorda A. Zeitlinger M. (2023). Individualized antimicrobial dose optimization: a systematic review and meta-analysis of randomized controlled trials. Clin. Microbiol. Infect. 29 (7), 845–857. 10.1016/j.cmi.2023.03.018 36965694

[B37] Sel E. K. Tufan B. Atagun G. K. Oguz V. A. Ozbek O. A. Gumustekin M. (2025). Association of vancomycin trough levels, AUC and AUC/MIC ratios with clinical outcomes in patients with enterococcal bacteremia: a prospective cohort study. BMC Infect. Dis. 25 (1), 958. 10.1186/s12879-025-11400-9 40731327 PMC12309136

[B38] Tanaka R. (2025). Pharmacokinetic variability and significance of therapeutic drug monitoring for broad-spectrum antimicrobials in critically ill patients. J. Pharm. Health Care Sci. 11 (1), 21. 10.1186/s40780-025-00425-6 40098009 PMC11912797

[B39] Tangvichitrerk P. Changpradub D. Hemapanpairoa J. Juntanawiwat P. Santimaleeworagun W. (2025). Impact of vancomycin area under the curve in early or later phase on efficacy and nephrotoxicity in patients with enterococcal bloodstream infections: a multicenter study. BMC Infect. Dis. 25 (1), 133. 10.1186/s12879-024-10399-9 39875832 PMC11773776

[B40] Tesfamariam N. S. Aboelezz A. Mahmoud S. H. (2024). The impact of augmented renal clearance on vancomycin pharmacokinetics and pharmacodynamics in critically ill patients. J. Clin. Med. 13 (8), 2317. 10.3390/jcm13082317 38673590 PMC11051385

[B41] Timsit J. F. Ruppé E. Barbier F. Tabah A. Bassetti M. (2020). Bloodstream infections in critically ill patients: an expert statement. Intensive Care Med. 46 (2), 266–284. 10.1007/s00134-020-05950-6 32047941 PMC7223992

[B42] Tochikura N. Matsumoto C. Iwabuchi S. Aso H. Fukushima S. Ootsuka S. (2024). Pharmacokinetic/pharmacodynamic analysis of vancomycin in patients with Enterococcus faecium bacteremia: a retrospective cohort study. Eur. J. Hosp. Pharm. 31 (5), 440–446. 10.1136/ejhpharm-2022-003672 36868850

[B43] Tong S. Y. C. Fowler V. G. Jr Skalla L. Holland T. L. (2025). Management of *Staphylococcus aureus* bacteremia: a review. JAMA. 334 (9), 798–808. 10.1001/jama.2025.4288 40193249 PMC12663922

[B44] Tsutsuura M. Moriyama H. Kojima N. Mizukami Y. Tashiro S. Osa S. (2021). The monitoring of vancomycin: a systematic review and meta-analyses of area under the concentration-time curve-guided dosing and trough-guided dosing. BMC Infect. Dis. 21 (1), 153. 10.1186/s12879-021-05858-6 33549035 PMC7866743

[B45] Udy A. A. Jarrett P. Lassig-Smith M. Stuart J. Starr T. Dunlop R. (2017). Augmented renal clearance in traumatic brain injury: a single-center observational study of atrial natriuretic peptide, cardiac output, and creatinine clearance. J. Neurotrauma 34 (1), 137–144. 10.1089/neu.2015.4328 27302851

[B46] Villanueva R. D. Talledo O. Neely S. White B. Celii A. Cross A. (2019). Vancomycin dosing in critically ill trauma patients: the VANCTIC study. J. Trauma Acute Care Surg. 87 (5), 1164–1171. 10.1097/TA.0000000000002492 31464871

[B47] Xiao Q. Zhang H. Wu X. Qu J. Qin L. Wang C. (2022). Augmented renal clearance in severe Infections-An important consideration in vancomycin dosing: a narrative review. Front. Pharmacol. 13, 835557. 10.3389/fphar.2022.835557 35387348 PMC8979486

[B48] Zhao J. Fan Y. Yang M. Liang X. Wu J. Chen Y. (2022). Association between augmented renal clearance and inadequate vancomycin pharmacokinetic/pharmacodynamic targets in Chinese adult patients: a prospective observational study. Antibiot. (Basel) 11 (7), 837. 10.3390/antibiotics11070837 35884091 PMC9312211

